# A fast and high performance multiple data integration algorithm for identifying human disease genes

**DOI:** 10.1186/1755-8794-8-S3-S2

**Published:** 2015-09-23

**Authors:** Bolin Chen, Min Li, Jianxin Wang, Xuequn Shang, Fang-Xiang Wu

**Affiliations:** 1 School of Computer Science, Northwestern Polytechnical University, 127 Youyi West Road, 710072, Xi'an, P.R. China; 2 School of Information Science and Engineering, Central South University, 410083, Changsha, P.R.China; 3 Division of Biomedical Engineering, University of Saskatchewan, 57 Campus Dr., S7N 5A9, Saskatoon, Canada; 4 Department of Mechanical Engineering, University of Saskatchewan, 57 Campus Dr., S7N 5A9, Saskatoon, Canada

**Keywords:** disease gene, Bayesian analysis, logistic regression, multiple data integration, feature vector

## Abstract

**Background:**

Integrating multiple data sources is indispensable in improving disease gene identification. It is not only due to the fact that disease genes associated with similar genetic diseases tend to lie close with each other in various biological networks, but also due to the fact that gene-disease associations are complex. Although various algorithms have been proposed to identify disease genes, their prediction performances and the computational time still should be further improved.

**Results:**

In this study, we propose a fast and high performance multiple data integration algorithm for identifying human disease genes. A posterior probability of each candidate gene associated with individual diseases is calculated by using a Bayesian analysis method and a binary logistic regression model. Two prior probability estimation strategies and two feature vector construction methods are developed to test the performance of the proposed algorithm.

**Conclusions:**

The proposed algorithm is not only generated predictions with high AUC scores, but also runs very fast. When only a single PPI network is employed, the AUC score is 0.769 by using *F*_2 _as feature vectors. The average running time for each leave-one-out experiment is only around 1.5 seconds. When three biological networks are integrated, the AUC score using *F*_3 _as feature vectors increases to 0.830, and the average running time for each leave-one-out experiment takes only about 12.54 seconds. It is better than many existing algorithms.

## Background

The term disease broadly refers to any condition that impairs normal conditions of part or all of an organism. Among various diseases, genetic disorders are those related to disfunction of one or multiple genes in the human genome. A genetic disorder may arise from or lead to mutations of one or more genes, or associate with over-/under expression of one or more genes [1]. This phenomenon is also a reflection of the module characteristic of real biological systems [2], where genes, proteins or other molecules often interact with each other to perform majority cellular processes [3-5]. Even disfunction of a single kind of gene may lead to disassembling some protein complexes or disturb a whole normal cellular pathway, thereby resulting in genetic disorders.

The issue of disease gene identification is to find those genetic disorder related genes, or called disease genes for short, for each specific genetic disease. Various kinds of evidence have shown that disease genes are not randomly distributed, but rather tend to lie close to each other in many biological networks if they are associated the same or similar diseases [1,2,6,7].

Various kinds of biological data sources have shown their power for identifying disease genes. Oti et al. [2] use several sets of protein-protein interaction (PPI) data to predict disease genes. They argue that the use of PPI data can greatly increase the prediction performance for disease gene identifications. Fraser et al. [8] investigate both yeast and human functional genomic data and argue that protein complexes contain valuable information which is helpful for detecting disease genes. Li et al. [9] investigate genetic diseases from a pathway based point of view. They find that individual pathways often enrich genes related to the same or similar diseases. Ma et al. [10] propose a combining gene expression and protein interaction (CGI) method to prioritize genes associated with a specific phenotype or trait. Ganegoda et al. [11] and Li et al. [12] use tissue-specific data together with PPI information to predict disease genes within individual tissues. Li et al. [13] also use Gene Ontology (GO) annotations to identify disease genes by combining topological features of PPI networks.

Besides different data sources, many different computational methods have also been employed for identifying disease genes. Lage et al. [14] propose a Bayesian method to analyze a phenome-interactome network. Wu et al. [7] use a linear regression method to calculate the concordance score between a PPI network and a phenotype network. A tool called CIPHER is developed to predict disease genes based on those concordance scores. Vanunu et al. [15] formulate a smoothness-related prioritization function in a PPI network, which predicts not only disease genes but also disease associated protein complexes. Zhang et al. [16] develop a Bayesian regression approach to explain similarities of disease phenotypes by using diffusion kernels of one or several PPI networks. Köhler et al. [17] propose a random walk with restart (RWR) algorithm to detect disease genes by using a global network distance measure and random walk analysis.

Among those algorithms, the RWR algorithm [17] often yields better performance than other algorithms in terms of the prediction accuracy and the running time. However, the RWR algorithm can only take a single network as the input. When multiple kinds of biological networks need to be integrated, the RWR algorithm can only simply merge them into a mixed network as the input. Although this strategy can integrate useful information from different data sources, it integrates noises from them as well. Predictions of the RWR algorithm from a mixed network do not always perform better than those from individual networks. To improve the data integration method, Chen et al. [18] define a data integration rank (DIR) score to select the most informative evidence among a set of data sources. Chen et al. [19,20] recently propose two improved Markov random field (MRF) algorithms, which can automatically assign weights to different data sources by using Gibbs sampling processes. They often yield better performance than those using only single data source, and the MRF algorithms are even more better than the the DIR method in terms of the prediction accuracy. However, the DIR algorithm is too time-consuming due to the calculating of a normalized similarity measure for all gene pairs, while the MRF algorithms spend more time to maintain a long Markov chain for every gene during the Gibbs sampling processes.

In paper [21], we have proposed a logistic regression based algorithm to reduce the computational time of the MRF algorithm. It directly formulates the issue of disease gene identification as a binary logistic regression problem by using similar feature vectors as the MRF algorithm. No Markov chains need to be maintained for all genes, which makes the algorithm runs very fast. However, the logistic regression based algorithm in [21] is only a single network based algorithm, and the feature vector construction method is limited to using information of only direct neighbors. In this paper, we propose a fast and high performance multiple data integration algorithm to generalize the logistic regression based algorithm in [21]. Two aspects of generalization are proposed: (1) the generalization of the feature vector construction method; and (2) the extension of the application scope for using multiple data integrations. To be more specific, we first theoretically introduce how binary logistic regression model is used to formulate the disease gene identification issue. Then, the feature vector construction method is generalized by using not only direct neighbors but also higher-order neighborhood information in a network. After that, the logistic regression based algorithm is extended to the multiple data integration case, where the parameters (weights) of different data sources can be tuned automatically. A prior probability estimation method is also proposed by using protein complex information, together with a validation method and evaluation criteria. The numerical experiments show that the proposed algorithm not only achieves high AUC score, but also runs very fast even in the multiple data integration case. It outperforms many existing algorithms for identifying human disease genes.

## Methods and materials

### Problem formulation

Let *H *be a bipartite graph consisting of two disjoint sets of vertices, where one set represents all known human genes *{g*_1_*, g*_2_*, . . . , g_N_}*, while the other set represents all known genetic diseases *{d*_1_*, d*_2_*, . . . , d_r_}*. The associations between those genes and genetic diseases can be obtained from either the Online Mendelian Inheritance in Man (OMIM) database [22] or similar databases.

Although a disease *d_k _*may associate with one or several genes, the number of all known disease genes *m *is much smaller than *N *. Hence, associations of most other genes are still not known and need to be analyzed. Without loss of generality, we can reorder the set of all human genes as a vector (*g*_1_, *g*_2_, . . . , *g*_N _), according to a set of given gene-disease associations, where *g*_*n*+1_, *g*_*n*+2_, . . . , *g*_*n*+*m *_are genes associated with at lease one known disease (disease genes), and *g*_1_, *g*_2_, . . . , *g*_*n *_are others. Here *N *= *n *+ *m*, and *n *is the number of all genes that are not known to associate with any diseases and they are called unknown genes in this paper.

For a specific disease *d_k_*, the issue of disease gene identification is to find a set of candidate genes which may have associations with *d_k_*. To achieve this, let $x^{k}=\left(x_{1}^{k},\;x_{2}^{k},...,x_{n+m}^{k}\right)$ be a vector of binary class labels (i.e. taking the value zero or one) defined on all genes, where $x_{i}^{k}=1$ represents gene *g_i _*being a disease gene of *d_k _*, and $x_{i}^{k}=0$ otherwise. Since we have to address each genetic disease one by one, we take *d*_*k *_for example, and ignore the superscript *k *in the vector *x*^*k *^for simplicity as *x *= (*x*_1_, *x*_2_, . . . , *x*_*n*+*m*_), hereafter. Therefore, the identification of disease genes is equivalent to find labels of *x_i _*for all unknown genes. Identification of disease genes for other diseases can be similarly conducted by changing *d_k _*to another disease.

**Figure 1:**
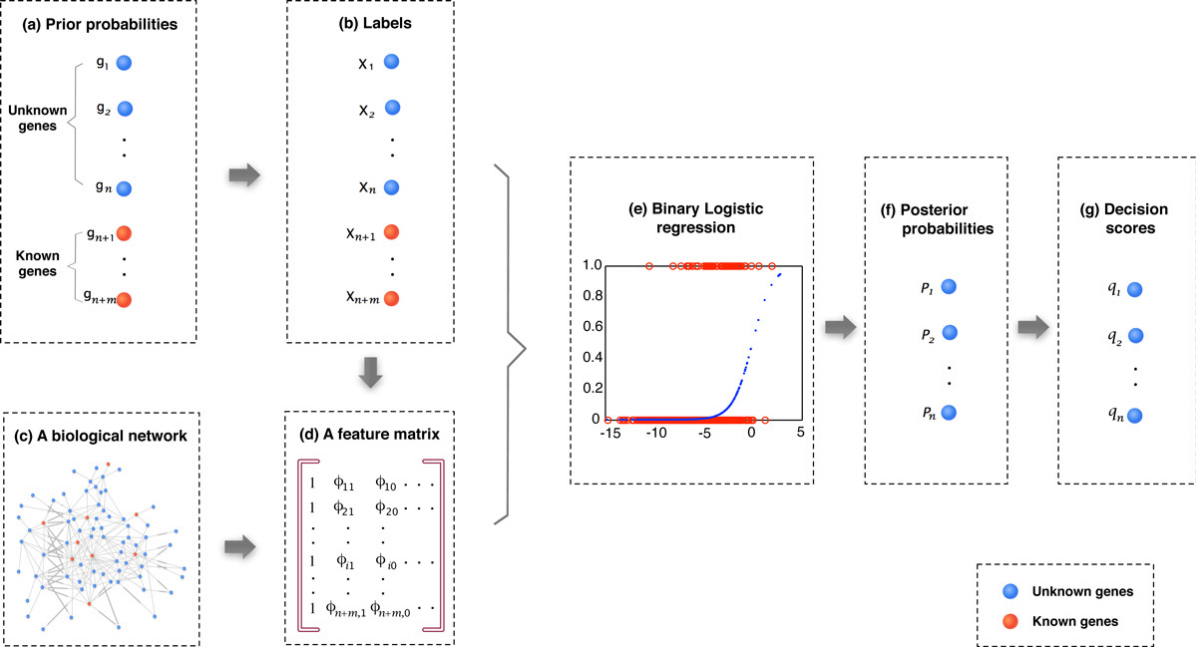
**The general idea of the proposed logistic regression based algorithm**. (a) A prior probability of each gene is first predefined. (b) The class label of each gene is then assigned according to its prior probability. (c) A biological network gives the neighborhood connections between individual genes. (d) A feature matrix is constructed based on the labels of individual vertices and the biological network. (e) A binary logistic regression is conducted by using class labels as categorical dependent variables and individual features as predictor variables. (f) A posterior probability is obtained from the binary logistic regression for each unknown genes. (g) The posterior probability is transformed into a decision score for each unknown genes. (a) - (f) make up the inference stage, while (g) is the decision stage.

In this paper, the issue of disease gene identification is formulated as a two-class classification problem by using Bayesian analysis and logistic regression. The conditional probability *p*(*x_i _*= 1*| *Φ) for each unknown gene is first calculated in an inference stage, and a decision score is then obtained according to this probability in a decision stage [23]. Here Φ represents the information used to make the inference, such as a vector of prior labels of *x*, the connectivity of the bipartite graph *H*, the neighborhood relationships of *g*_1_*, g*_2_*, . . . , g_N _*, and the similarity relationship between *d*_1_*, d*_2_*, . . . , d_r _*. The flow diagram of the proposed algorithm is depicted in Figure 1.

### Prior label estimation

The logistic regression based algorithm needs a vector of prior labels for *x*. For those known disease genes, one can directly assign 1 or 0 according to the known gene-disease associations. For those unknown disease genes, a prior probability of each gene get the label 1 should be first estimated.

The simplest way is to assign the prior probability as 0 for all unknown genes. The prediction results in this case is denoted as *P*_0 _hereafter.

However, one can make it better by using additional prior information, such as protein complex data, to estimate prior probabilities for unknown genes. This is not only due to the fact that they are naturally available from various databases, but also due to their capability to describe the module characteristic of disease genes. If a disease is resulted from the disfunction of a protein complex, then any component of the complex should associate with the disease with a high probability.

Similar to the method used in [19,20], if a gene *g_i _*encodes a protein in a complex, then let

(1) $${\hat{p}}_{i}=\frac{A}{B}$$

be its prior probability, where *A *is the number of disease genes of the specific disease in the complex, and *B *is the number of all disease genes in the complex. If *g_i _*appears in multiple protein complexes, we use the maximum value as its prior probability. If *g_i _*does not belong to any protein complex, let

(2) $${\hat{p}}_{i}=\frac{C}{D}$$

be its prior probability, where *C *is the number of all currently known disease genes of the specific disease, and *D *is the total number of genes in human genome.

Once a prior probability ${\hat{p}}_{i}$ is estimated for *g_i_*, its prior label of ${\hat{x}}_{i}$ can be obtained as follows. First, generate a random number following the standard uniform distribution. If the value of the random number is large than ${\hat{p}}_{i}$, then assign 0 as the prior label for *g_i_*. Otherwise, assign 1 as the prior label for *g_i_*. Repeat this step for all unknown genes, one can obtain prior labels for all of them. The prediction results generated by using those prior labels is denoted as *P_c _*hereafter.

### Logistic regression

For a two-class classification problem, each gene is labelled with either 1 or 0. A vector of all binary values of *x *is called a *configuration*. In the previous MRF algorithm [19,20], the configuration *x *is formulated as an MRF which follows a Gibbs distribution. However, the Markovianity characteristic of the MRF model makes it only considering direct neighbors to construct feature vectors, which limits the capability of the method to use other topological attributes in a biological network. It is also very time consuming to maintain Markov chains for all unknown genes. In [21], we have introduced a logistic regression based algorithm to directly estimate the configuration by using the same feature vectors. However, the application of the logistic regression based algorithm is still limited to single biological network. A multiple data integration method should be further investigated. To generalize the formulation of feature vectors by using other topological attributes and extend its applicability to multiple data integration, we propose an improved logistic regression based algorithm in this study as follows.

Let *C*_1 _be a set of genes with label 1 and *C*_0 _be a set of genes with label 0. Suppose the following four kinds of probabilities are given: the class-conditional densities *p*(*x|C*_1_) and *p*(*x|C*_0_), which indicate the probability of the configuration *x *conditional on *C*_1 _and *C*_0_, respectively, and the class prior densities *p*(*C*_1_) and *p*(*C*_0_), which indicate the prior probability of genes in *C*_1 _and *C*_0 _being labelled with 1 and 0, respectively.

According to the Bayes' rule, the posterior probabilities of those genes in *C*_1 _that are labelled with 1 can be described as a logistic sigmoid function [23,24]

(3) $$p\left(C_{1}|x\right)=\frac{p\left(x|C_{1}\right)p\left(C_{1}\right)}{p\left(x|C_{1}\right)p\left(C_{1}\right)+p\left(x|C_{0}\right)p\left(C_{0}\right)}=\frac{e^{t}}{e^{t}+1}$$

and the posterior probabilities of those genes in *C*_0 _that are labelled with 0 can be similarly written as

(4) $$p\left(C_{0}|x\right)=\frac{p\left(x|C_{0}\right)p\left(C_{0}\right)}{p\left(x|C_{1}\right)p\left(C_{1}\right)+p\left(x|C_{0}\right)p\left(C_{0}\right)}=\frac{1}{e^{t}+1}$$

where the variable *t *is defined as

(5) $$t=\text{ln}\frac{p\left(x|C_{1}\right)p\left(C_{1}\right)}{p\left(x|C_{0}\right)p\left(C_{0}\right)},$$

which is related to the four kinds of probabilities.

Although *t *is often unavailable for a real problem, under general assumptions [23], *t *can be formulated as a function of different features *t *= *f *(*·*) associated with the integrated networks. To be more specific, let  x be a prior configuration of all human genes and *f *be a function. For any given gene *g_i_*, let *ϕ_i _*be the feature vector of *g_i _*that is related to the prior configuration $\hat{x}$. The posterior probability that the specific gene *g_i _*has label 1 and 0 are

(6) $$p\left(x_{i}=1|\phi_{i},f\right)=\frac{\text{exp}\left(f\left(\phi_{i}\right)\right)}{\text{exp}\left(f\left(\phi_{i}\right)\right)+1},$$

and

(7) $$p\left(x_{i}=0|\phi_{i},f\right)=\frac{1}{\text{exp}\left(f\left(\phi_{i}\right)\right)+1}.$$

respectively. Note that the sum of these two probabilities (6) and (7) must equal to 1 in this two-class classification problem. A linear function *f *(*ϕ_i_*) = *w^T ^ϕ_i _*with variables (feature vectors) *ϕ_i _*and coefficients (parameters) *w *is the most commonly used function to ensure the calculation of the posterior probability not too complex.

The key step of the proposed algorithm is the construction of feature vectors. In the previous methods [19,21], the numbers of direct neighbors that connects to disease genes and non-disease genes are employed as the feature vector for each gene. Take *g_i _*for example, its feature vector can be written as

(8) $$\phi_{i}={\left(1,\phi_{i1},\phi_{i0}\right)}^{T},$$

where *ϕ*_*i*1 _and *ϕ*_*i*0 _are the number of direct neighbors of *g_i _*that connected to vertices with labels 1 and 0, respectively. It is a three dimensional vector, where the first element represents the constant term. All feature vectors of individual genes together form a feature matrix as

(9) $$F_{1}={\left[\begin{array}{ccc} 1 & \phi_{11} & \phi_{10}\\ 1 & \phi_{21} & \phi_{20}\\ \vdots & \vdots & \vdots \\ 1 & \phi_{N1} & \phi_{N0} \end{array}\right]}_{{}_{N\times 3}}$$

where *N *is the number of all human genes. The corresponding parameters are *w *= (*w_0_, w_1_, w_2_*)*^T^*. Predictions generated by using (9) are denoted as *F*_1 _hereafter.

In this study, two extended feature vector construction methods are proposed as follows. Firstly, in a single biological network, not only the number of direct neighbors of *g_i_*, but also the number of its second order neighbors are employed to construct the feature vector as

(10) $$\phi_{i}={\left(1,\phi_{i1},\phi_{i0},{\phi^{\prime }}_{i1},{\phi^{\prime }}_{i0}\right)}^{T}$$

where *ϕ*_*i*1 _and *ϕ*_*i*0 _are the numbers of direct neighbors of *g_i _*connected to vertices with labels 1 and 0, respectively, and $\phi_{i1}^{\prime }$and $\phi_{i0}^{\prime }$ are the numbers of the second order neighbors of *g_i _*connected to vertices with labels 1 and 0, respectively. The contribution of those indirect neighbors has been investigated for predicting disease genes in [20,25,26]. The feature matrix in this situation can be written as

(11) $$F_{2}={\left[\begin{array}{ccccc} 1 & \phi_{11} & \phi_{10} & {\phi^{\prime }}_{11} & {\phi^{\prime }}_{10}\\ 1 & \phi_{21} & \phi_{20} & {\phi^{\prime }}_{21} & {\phi^{\prime }}_{20}\\ \vdots & \vdots & \vdots & \vdots & \vdots \\ 1 & \phi_{N1} & \phi_{N0} & {\phi^{\prime }}_{N1} & {\phi^{\prime }}_{N0} \end{array}\right]}_{N\times 5.}$$

The corresponding parameter vector *w *= (*w*_0_*, w*_1_*, w*_2_*,w*_3_*, w*_4_)*^T ^*is a five dimensional vector. Predictions generated by using (11) are denoted as *F*_2 _hereafter.

Secondly, in the multiple data integration situation, suppose there are *l *biological networks. Let $\phi_{i1}^{j},\phi_{i0}^{j}$ be the number of direct neighbors of *g_i _*connected to vertices with labels 1 and 0 in the *j^th ^*network, respectively. The feature vector obtained from those *l *networks

(12) $$\phi_{i}={\left(1,\phi_{i1}^{1},\phi_{i0}^{1},...,\phi_{i1}^{l},\phi_{i0}^{l}\right)}^{T}$$

is a 2*l *+ 1 dimensional vector. All those feature vectors together form a feature matrix as

(13) $$F_{3}={\left[\begin{array}{cccccc} 1 & \phi_{11}^{1} & \phi_{10}^{1} & \cdots \; & \phi_{11}^{l} & \phi_{10}^{l}\\ 1 & \phi_{21}^{1} & \phi_{20}^{1} & \cdots \; & \phi_{21}^{l} & \phi_{20}^{l}\\ \vdots & \vdots & \vdots & \cdots \; & \vdots & \vdots \\ 1 & \phi_{N1}^{1} & \phi_{N0}^{1} & \cdots \; & \phi_{N1}^{l} & \phi_{N0}^{l} \end{array}\right]}_{N\times \left(2l+1\right).}$$

The corresponding parameter vector *w *= (*w*_0_*, w*_1_*, w*_2_*,. . . , w*_2*l−*1_*, w*_2*l*_)*^T ^*is a 2*l *+ 1 dimensional vector, and *N *is the number of all human genes. Predictions generated from (13) by integrating multiple networks is denoted as *F*_3 _hereafter.

### Parameter estimation

Parameter estimation can be conducted on a training set consists of known disease genes, where known genes associated with *d_k _*are labelled with 1 and known genes associated with other diseases are labelled with 0. However, as we discussed in [19,21], the exclusion of most unknown genes reduces the number of vertices with label 0 significantly, thereby making the estimation of parameters inaccurate. Predictions from those inaccurate parameters are unreliable in disease gene identification.

It is noteworthy that the majority of human genes should not be disease genes associated with *d_k_*. Hence, the inclusion of all unknown genes with prior labels as the training set will make the training set more reasonable, where the number of vertices with label 0 is significantly increased, while the number of vertices with label 1 does not change too much. Such a training set, which consists of both known genes and unknown genes, has proved its powerful and efficient to estimate meaningful parameters in [19-21].

Given a prior configuration $\hat{x}$ for all vertices, a maximum-likelihood estimation (MLE) method can be employed to estimate the parameter vector *w*. The likelihood function can be written as

(14) $$L\left(w;x_{1},x_{2},...,x_{N}\right)=\overset{N}{\underset{i=1}{\prod }}p\left(x_{i}|\phi_{i,}f\right).$$

where *x_i _*is the label of *g_i_, ϕ_i _*is its feature vector that is calculated according to $\hat{x}$, *f *is a linear function of *ϕ_i _*with the form as *f *(*ϕ_i_*) = *w^T ^ϕ_i_*, and *N *is the number of all human genes. The log likelihood of (14) is

ln L (*w*; *x*_1_*, x*_2_*, . . . , x_N _*)

(15) $$=\overset{N}{\underset{i=1}{\sum }}\left[x_{i}w^{T}\phi_{i}-\text{ln}\left(1+\text{exp}\left(w^{T}\phi_{i}\right)\right)\right].$$

The log likelihood (15) is a convex function [27]. Hence, we can find an unique global optimal solution by solving a convex optimization problem. In this study, the standard MATLAB function *fminunc*() is employed to find a numerical solution of (15) (by calculating the minimum of *− *ln L (*w*; *x*_1_*, x*_2_*, . . . , x_N_*)). The the initial value of *w *is simply set as zero for the *fminunc*() function.

### Decision score and evaluation methods

The logistic regression based algorithm returns a set of posterior probabilities during the inference stage. One can directly use those probabilities to make decisions in the following decision stage. However, the posterior probabilities do not always work well due to the hardness to set a threshold for a genetic disease. Inspired by the DIR method [18], we propose to use a percentage value of a posterior probability as the decision score for each gene. The decision score is calculated as follows

(16) $$q_{i}=\frac{\left|\left\{j|p_{i}\geq p_{j}\right\}\right|}{n},i=1,2,...,n$$

where *{p*_1_*, p*_2_*, . . . , p_n_} *is the posterior probabilities of individual unknown genes, and *q_i _*is the top percentage value of *p_i _*among all those posterior probabilities. A candidate gene is more likely to be associated with *d_k_*, if its decision score is larger than majority of others.

To evaluate the performance of the proposed algorithm, the leave-one-out cross validation paradigm is employed by using above decision scores. The receiver operating characteristic (ROC) curve is employed as one of the evaluation criteria, which shows the relationship between the true positive rate (TPR) and the false positive rate (FPR) by varying a threshold for determining positives. The area under the ROC curve (AUC) is employed to show the overall performance of algorithms.

The positive control genes are those known disease genes associated with *d_k_*. For those negative control genes, although they are indispensable to calculate false positives and true negatives, it is generally hard to obtain a true negative dataset [28]. In this study, the negative control genes are randomly selected from known disease genes that do not associate with *d_k_*. Since those genes have been widely studied as disease genes for other genetic diseases, it is less likely for them to be disease genes for a different specific disease. If there are *s *known disease genes associated with *d_k_*, we randomly select $\left\lfloor \frac{s}{2}\right\rfloor$ such genes as a negative control set. Each gene belonging to the negative control set is also validated by using the leave-one-out cross validation paradigm.

The proposed algorithm is compared with four previous algorithms: (1) the initial logistic regression based algorithm proposed in [21]; (2) the RWR algorithm proposed in [17]; (3) the MRF algorithm proposed in [19]; and (4) the DIR algorithm proposed in [18]. The first algorithm is applicable to a single network. The second and the third algorithms are applicable to both single network and multiple data integration. The fourth algorithm works only for multiple data integration. All those algorithms identify disease genes with high prediction performance and they work better than many previous methods [17-19,21].

### Algorithm

The step-by-step description of the proposed logistic regression based algorithm is given as follows.

**Input: **The vector of all human genes (*g*_1_, . . . , *g*_*n*+*m*_), where (*g*_1_*, . . . , g_n_*) are unknown genes, and (*g*_*n*+1_, . . . , *g*_*n*+*m*_) are known genes; *l *integrated biological networks *G*_1_*, G*_2_*, . . . , G_l_*; a set of protein complexes; and a set of gene-disease associations.

**Output: **The vector of decision score for each unknown gene for each disease.

1: For a specific disease *d_k _*, calculate prior probabilities for all human genes, where the prior probability of unknown genes ${\hat{p}}_{1},...,{\hat{p}}_{n}$ are calculated according to (1) and (2).

2: For each known gene *g*_*n*+*i*_, *i *= 1*, . . . , m*, if *g*_*n*+*i *_is known to be associated with *d_k _*, let ${\hat{p}}_{n+i}=1.$ Otherwise, let ${\hat{p}}_{n+i}=0.$

3: Assign prior labels $\hat{x}=\left({\hat{x}}_{1},{\hat{x}}_{2},...,{\hat{x}}_{n},{\hat{x}}_{n+1},...,{\hat{x}}_{n+m}\right)$ for all genes according to the prior probabilities $\left({\hat{p}}_{1},...,{\hat{p}}_{n+m}\right),$ respectively.

4: Calculate the feature vector *ϕ_i _*for each *g_i _*according to the integrated biological networks and $\hat{x}$.

5: Estimate parameters  ŵ by maximizing the log likelihood ln $L\left(w;x_{1},x_{2},...,x_{N}\right)$ in (15) based on $\hat{x}$ and *ϕ_i_, i *= 1*, . . . , n *+ *m*. A binary logistic regression is performed here by taking the vector $\hat{x}$ as the categorical dependent variables and those label-related feature vectors *ϕ_i _*as predictor variables. Here *i *= 1*, . . . , N*.

6: Calculate the posterior probability *p*_1_*, . . . , p_n _*for each unknown gene according to (6) by using  ŵ and *ϕ_i_*.

7: Calculate the decision scores *q*_1_*, . . . , q_n _*according to (16).

8: Repeat all the steps for another disease until every disease is checked.

## Results and discussion

### Data sources

We use the same datasets as [19] in order to directly compare with previous methods. To be more specific, gene-disease associations are collected from the Morbid Map list of the Online Mendelian Inheritance in Man (OMIM) [22]. Since a disease generally only associates with a few disease genes, it is hard to perform a logistic regression based on such small amount of positive samples. Hence, merging similar diseases into a disease class, and identifying disease genes associated with the disease class can circumvent this problem to some extent. Goh et al. [1] manually classify all diseases in OMIM into 22 primary disease classes. The dataset contains 1284 genetic diseases and 1777 disease genes. In this study, we use twelve disease classes that consist of 815 genes to test the performance of the proposed algorithm.

The PPI dataset is derived from the database of HPRD (Release 9) [29]. Duplicated edges between the same pair of vertices and self-loop edges are deleted. The final PPI network consists of 9465 vertices and 37039 edges. Two another PPI datasets are derived from the database of BioGrid (Release 3.2.108) [30] and the database of IntAct (downloaded on Jan 26, 2014) [31], respectively, which are used to select edges of biological networks.

The pathway datasets are obtained from the database of KEGG [32], Reactome [33], PharmGKB [34], and PIN [35]. There are 280, 1469, 99 and 2679 pathways in those datasets, respectively. The total number of proteins/genes consisting of those pathways is 8614. A pathway co-existing network is constructed by taking individual proteins/genes as vertices. Edges are constructed between two vertices, if they co-exist in any pathway.

The human gene expression profiles are obtained from BioGPS (GSE1133) [36,37], which contains 79 human tissues in duplicates, measured using the Affymetrix U133A array. Pairwise Pearson correlation coefficients (PCC) are calculated. A pair of genes are linked by an edge if the PCC value is large than 0.5, similar to the method used in [1,18] to construct the gene co-expression network.

The human protein complexes are collected from the database of CORUM [38] and PCDq [39]. There are 1677 and 1103 protein complexes in datasets with at least two proteins, respectively. There are in total 3881 proteins in those protein complexes.

In summary, three kinds of biological networks are constructed and all protein (or gene) IDs are mapped onto the form of gene symbol. In order to test the performance of multiple data integration of our method, we selected those vertices that appear at least four times in all five biological networks (three PPI networks, a pathway co-existing network and a gene co-expression network). The final datasets consist of 7311 human genes, 815 out of which are known associated with 12 disease classes. The details of those datasets used in this study can be found in the "Availability of supporting data" section.

### Comparisons between different priors

If there is no prior information available for the application of the proposed algorithm, zero prior *P*_0 _still works in most situations. However, if there is general prior information available in practice (such as the protein complex information), the proposed algorithm should work better than that using *P*_0_.

**Figure 2:**
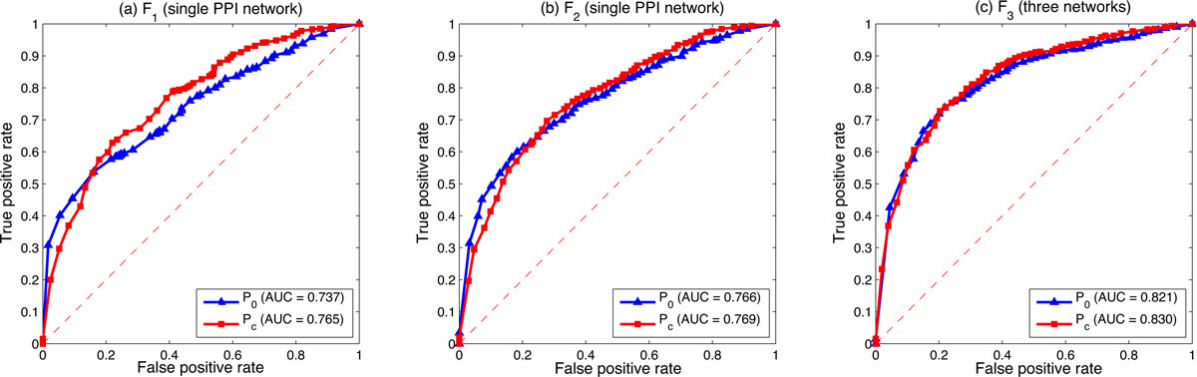
**Comparisons between different priors of the logistic regression based algorithm by using three kinds of feature vectors**. (a) The ROC curve of the proposed algorithm by using *F*_1 _on the single HPRD PPI network. (b) The ROC curve of the proposed algorithm by using *F*_2 _on the single HPRD PPI network. (c) The ROC curve of the proposed algorithm by using *F*_3 _by integrating three biological networks: the HPRD PPI network, the pathway co-existing network and the gene co-expression network. AUC values are listed in parentheses.

Figure 2 compares the logistic regression based algorithm by using either the zero prior *P*_0 _or the protein complex prior *P_c_*. We can see from Figure 2 that *P_c _*always works better than *P*_0 _in all three kinds of feature vectors in terms of the AUC score. The highest improvement is achieved when *F*_1 _is employed, where the AUC score increases from 0.737 to 0.765. There is only slight improvement when *F*_3 _is employed in multiple data integration, where the AUC score increases from 0.821 to 0.830. This may due to the fact that *F*_1 _using *P*_0 _achieves the lowest prediction AUC score for identifying disease genes. It has the highest potential to be improved. While *F*_3 _using *P*_0 _in the multiple data integration already achieves a very high AUC score. There is only a little room for it to be further improved by using additional prior information.

Although the improvement of the protein complex information is not so significant for *F*_2 _and *F*_3_, the increased AUC score still indicates that additional knowledge is helpful for improving the prediction performance. This characteristic makes the proposed algorithm very promising, since it is flexible in terms of the usage of different prior information. Any prior knowledge related to gene-disease associations can be employed to estimate the prior labels.

### Comparisons between different feature vectors

**Figure 3:**
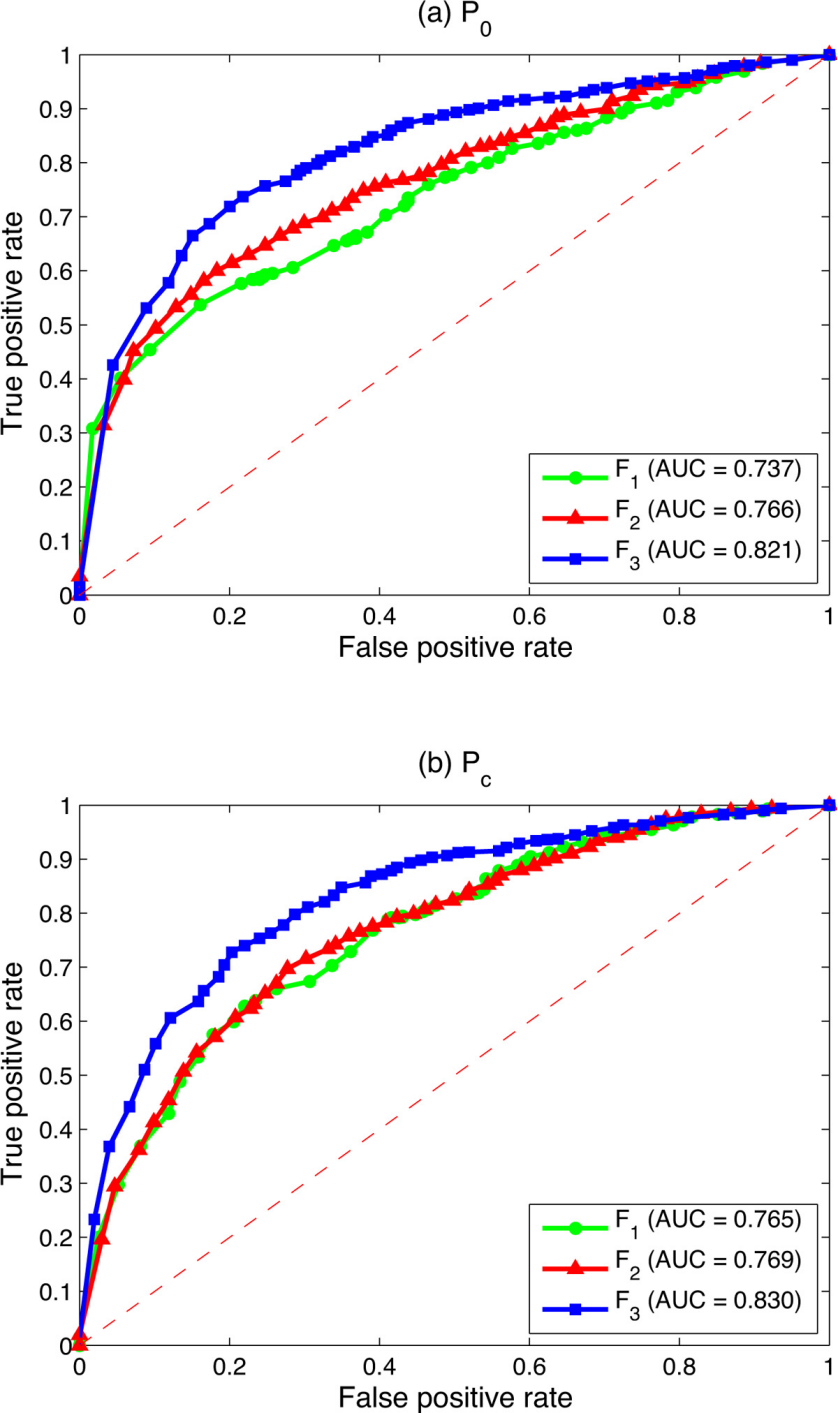
**Comparisons between different feature vectors of the logistic regression based algorithm**. (a) The ROC curve of different feature vectors by using *P*_0_. (b) The ROC curve of different feature vectors by using *P_c_*. AUC values are listed in parentheses.

Figure 3 compares the logistic regression based algorithm by using different feature vectors. *F*_1 _and *F*_2 _are tested on the single HPRD PPI network, and *F*_3 _is tested by integrating the following three biological networks: (1) the HPRD PPI network, (2) the pathway co-existing network and (3) the gene co-expression network. They are the same experimental results as Figure 2 shows, but from a different point of view.

We can see from Figure 3 that *F*_3 _achieves the highest AUC score in both *P*_0 _and *P_c_*, while *F*_1 _always obtains the lowest AUC score. In the zero prior situation *P*_0_, *F*_1 _reaches the AUC score at only 0.737, *F*_2 _on the same single PPI network reaches that at 0.766, while *F*_3 _by integrating three networks achieves the AUC score at 0.821. In the protein complex prior situation *P_c_*, the AUC score of *F*_1 _is 0.765. It increases to 0.769 by using *F*_2 _on the same single PPI network, and it continually rises to 0.830 by using *F*_3 _in the multiple data integration. Both *F*_2 _and *F*_3 _proposed in this study work better than the initial feature vector *F*_1_.

### Comparing with previous algorithms

To test the efficiency of the proposed algorithm, four previous algorithms are employed as comparison in either single network or multiple data integration. The initial logistic regression works only in single network, while the DIR algorithm works only in multiple data integration.

**Figure 4:**
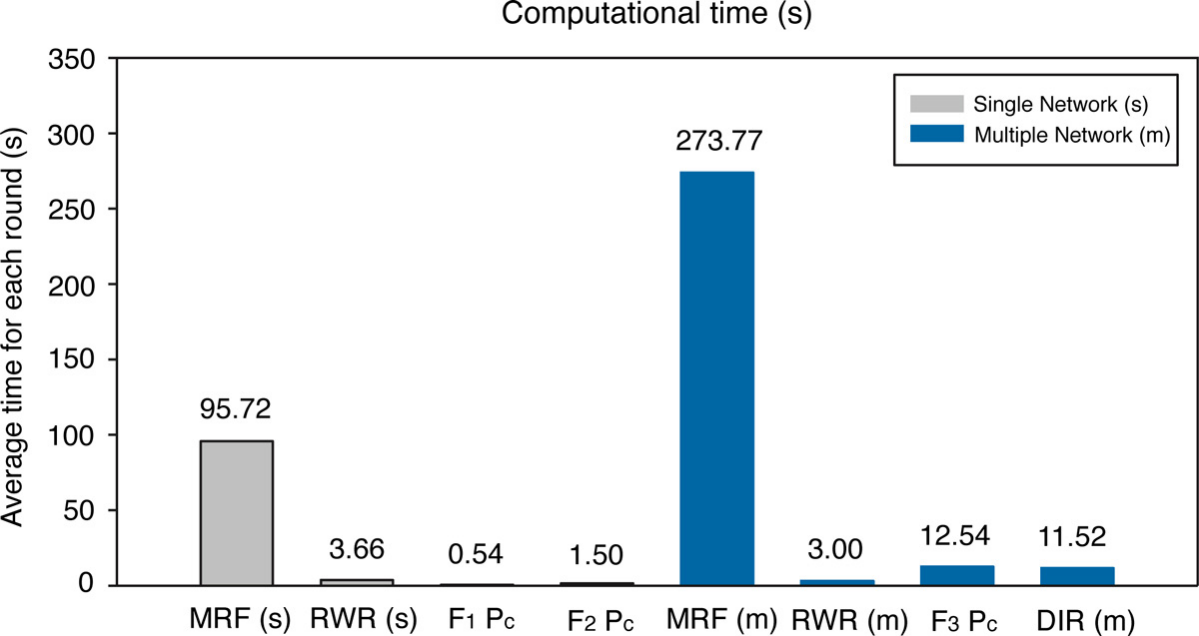
**Comparison of the computational time among different algorithms**. The grey bars illustrate the average time of different algorithms that work on the single HPRD PPI network. From left to right, they are the average computational time of the MRF algorithm, the RWR algorithm, the initial proposed algorithm by using the *F*_1_*P_c _*and the proposed logistic regression based algorithm by using *F*_2_*P_c_*, respectively. The blue bars illustrate the average time of different algorithms by integrating three biological networks. From left to right, they are the average computational time of the MRF algorithm, the RWR algorithm, the proposed logistic regression based algorithm by using the *F*_3_*P_c _*and the DIR algorithm, respectively. The number above each bar gives the average time (by second) for each leave-one-out experiment.

The comparison is first conducted in terms of the computational time. All those tests are conducted on a Windows 7 professional computer (Inter(R) Core(TM) i7 CPU, 3.07 GHz, 8.0 GB RAM, 64-bit OS). The MATLAB version is 7.10.0.499 (R2010a), 64-bit (win 64). Each algorithm is evaluated by using the leave-one-out cross validation paradigm, where each known gene is left out once. The probabilities of all unknown genes (include the left out one) are calculated by using each algorithm. All algorithms are conducted on the same datasets and the same computational conditions. Figure 4 illustrates the average computational time for each leave-one-out experiment among different algorithms.

We can see from Figure 4 that the MRF algorithm is the slowest algorithm. A leave-one-out experiment spends around 95.72 seconds for the single network, and it increases to about 273.77 seconds when three biological networks are integrated. The initial logistic regression based algorithm *F*_1 _runs very fast. It only spends approximately 0.54 seconds in the single network. The improved logistic regression based algorithms *F*_2 _and *F*_3 _also runs very fast. It only takes around 1.5 seconds when *F*_2 _is used, and it increases to about 12.54 seconds when three biological networks are integrated by using *F*_3_, which is almost the same as the DIR algorithm (11.52 seconds). The RWR algorithm also runs very fast, and it does not vary too much in both situations. It is due to the fact that the RWR algorithm uses the mixed network as input. No matter how many networks are integrated, it combines them together as a single mixed network. Hence, the number of integrated networks does not affect the computational time significantly.

**Figure 5:**
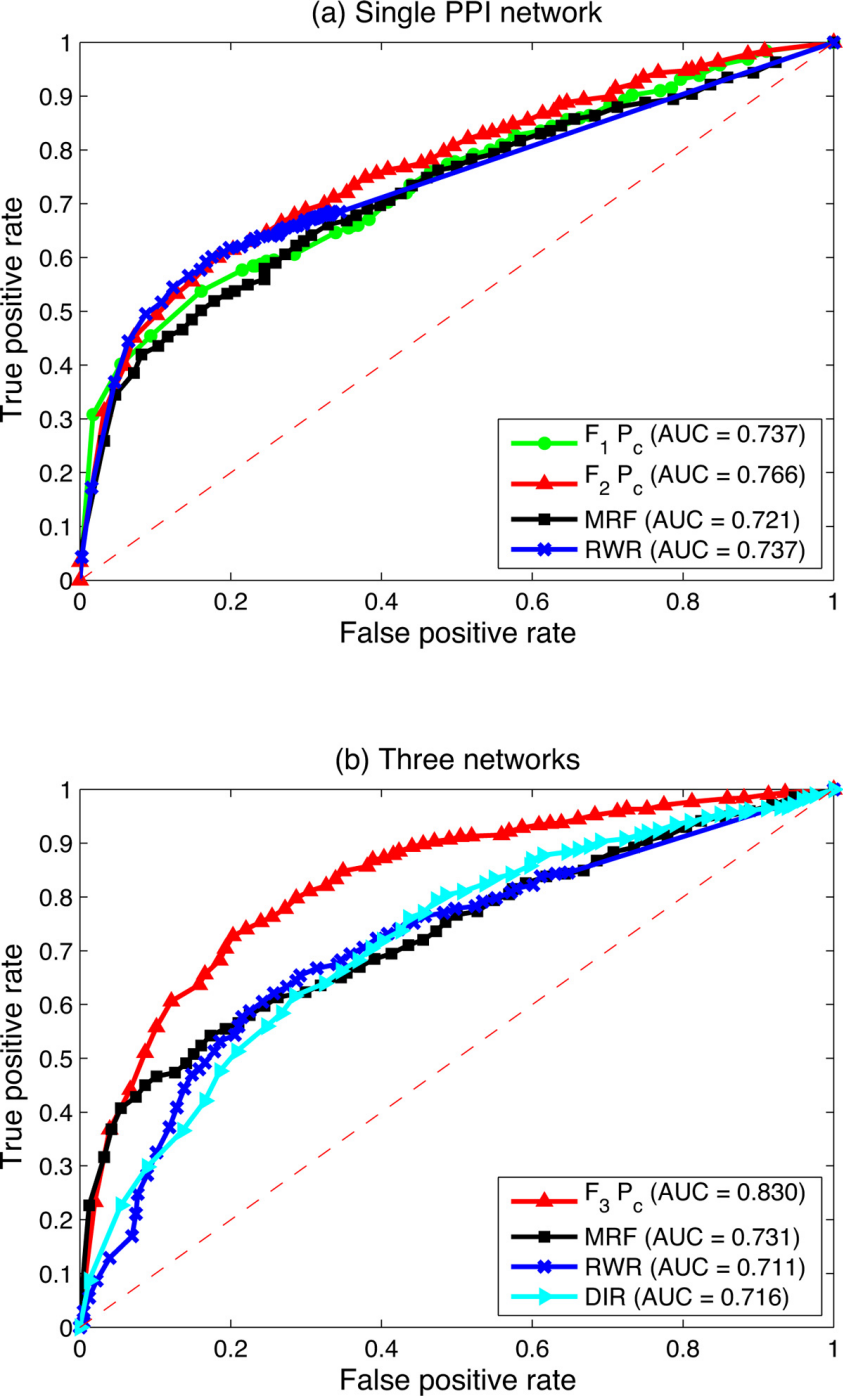
**ROC curves of cross-validation results of the proposed logistic regression based algorithm and three previous methods**. (a) The ROC curves of different algorithms conducted on the single HPRD PPI network. (a) The ROC curves of different algorithms conducted on the integrated three biological networks: the HPRD PPI network, the pathway co-existing network and the gene co-expression network. AUC values are listed in parentheses.

The comparison is then conducted in terms of the AUC scores. When only the single HPRD PPI network is employed, as illustrated in Figure 5(a), the proposed logistic regression based algorithm using *F*_2 _works better than all of the previous single network based algorithms. The AUC score is 0.766, which achieves 2.9%, 4.5% and 2.9% improvements compared with the initial logistic regression based algorithm using *F*_1_, the MRF algorithm and the RWR algorithm, respectively. When three biological networks are employed, as illustrated in Figure 5(b), the proposed logistic regression based algorithm using *F*_3 _achieves the highest AUC score among all these multiple data integration algorithms. The AUC score is 0.830 when protein complex prior *P_c _*is used, which is 9.9%, 11.9% and 11.4% improvements compared with the MRF algorithm, the RWR algorithm and the DIR algorithm under the same situation, respectively.

**Figure 6:**
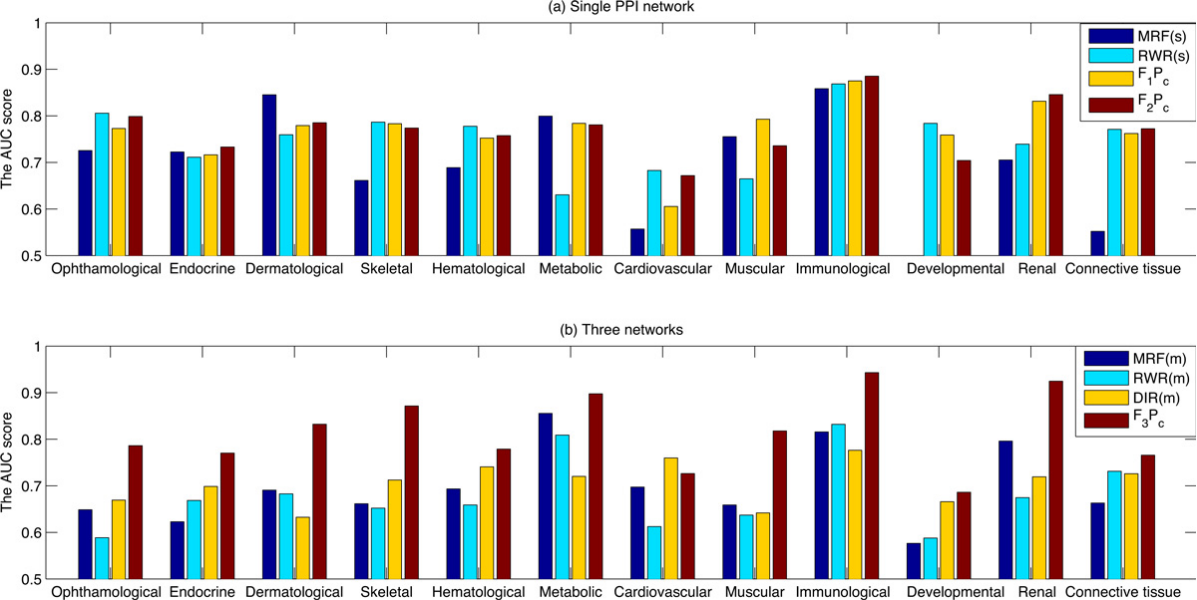
**The AUC scores of different prediction algorithms for each of the 12 disease classes**. (a) The AUC scores of different algorithms conducted on the single HPRD PPI network. (b) The AUC scores of different algorithms conducted on three biological networks: the HPRD PPI network, the pathway co-existing network and the gene co-expression network.

The comparison is finally conducted in terms of the AUC scores for each of the 12 disease classes. It can be seen from the Figure 6(a) that the proposed logistic regression based algorithm (*F*_2_*P_c_*) is the most stable algorithm in the single network. Its AUC score is larger than the other three algorithms in many cases. When three biological networks are integrated, the proposed logistic regression based algorithm (*F*_3_*P_c_*) achieves the highest AUC score in all cases, which makes the algorithm very promising in terms of multiple data integration.

## Conclusions

In this paper, we have proposed an improved logistic regression based algorithm to identify disease genes by using either a single network or multiple networks. A Bayesian analysis method is first used to formulated the disease gene identification issue as a two-class classification problem. A binary logistic regression model is then employed to calculate the posterior probability of each unknown gene obtained the label 1. Parameters of the model are estimated based on the whole gene set, and the final decision scores are obtained by using the percentage values of individual posterior probabilities.

Compared with previous algorithms, the proposed logistic regression based algorithm not only runs fast, but also generates predictions with high AUC scores. It only takes around 1.50 seconds in the single PPI network, and the AUC score is larger than all of the three single network based competing algorithms. Although the running time for the multiple networks is a little longer than the RWR algorithm and the DIR algorithm, it is still comparable, and the AUC score of the proposed algorithm is much better than those two algorithms. Compared with the MRF algorithm, the computational time has been significantly reduced, while the predictive performance becomes much better in terms of the AUC score. The best AUC score of the proposed algorithm is 0.766 in the single network, and it increases to 0.830 if three networks are integrated. The high prediction performance and the short computation time make the proposed algorithm very promising for identifying human disease genes.

## Availability of supporting data

The Matlab code of the proposed algorithm with data can be found in *https://www.dropbox.com/s/bs0ekmu718u4sea/Package15.zip*

## List of abbreviations

AUC, area under the ROC curve; CGI, combining gene expression and protein interaction; DIR, data integration rank; FPR, false positive rate; MRF, Markov random field; OMIM, online Mendelian inheritance in man; PCC, Pearson correlation coefficient; PPI, protein-protein interaction; ROC, receiver operating characteristic; RWR, random walk with restart; TPR, true positive rate.

## Competing interests

The authors declare that they have no competing interests.

## Authors' contributions

FXW initiated this study, FXW and BC designed algorithms and experiments. BC performed the experiments, analyzed the results, and drafted the manuscript. FXW, ML, JW and XS revised the manuscript. All authors have read and approved the final manuscript.

## Declarations

The publication costs for this article were supported by the Natural Sciences and Engineering Research Council of Canada (NSERC), the National Natural Science Foundation of China (Grant No. 61332014 and 61370024), and the fundamental research program of the Northwestern Polytechnical University (Grant No. G2015KY0104 and G2015KY0302).

This article has been published as part of BMC *Medical Genomics *Volume 8 Supplement 3, 2015: Selected articles from the IEE International Conference on Bioinformatics and Biomedicine (BIBM 2014): Medical Genomics. The full contents of the supplement are available online at http://www.biomedcentral.com/bmcmedgenomics/supplements/8/S3.
